# Regulation of the EGFR Pathway by HSP90 Is Involved in the Pathogenesis of Cushing’s Disease

**DOI:** 10.3389/fendo.2020.601984

**Published:** 2021-01-18

**Authors:** Yue Shen, Chenxing Ji, Xuemin Jian, Juan Zhou, Qilin Zhang, Nidan Qiao, Yichao Zhang, Xuefei Shou, Xiang Zhou, Zengyi Ma

**Affiliations:** ^1^ Department of Neurosurgery, Huashan Hospital, Shanghai Medical College, Fudan University, Neurosurgical Institute of Fudan University, Shanghai Clinical Medical Center of Neurosurgery, Shanghai Key Laboratory of Brain Function Restoration and Neural Regeneration, Shanghai, China; ^2^ Shanghai Pituitary Tumor Center, Shanghai, China; ^3^ Shanghai Jiao Tong University School of Medicine, Bio-X Institutes, Key Laboratory for the Genetics of Developmental and Neuropsychiatric Disorders (Ministry of Education), and the Collaborative Innovation Center for Brain Science, Shanghai Jiao Tong University, Shanghai, China

**Keywords:** epidermal growth factor receptor, Cushing’s disease, Hsp90, 17-N-allylamino-17-demethoxygeldanamycin/tanespimycin, pathogenesis

## Abstract

**Purpose:**

To investigate the role of heat-shock protein Hsp90 in adrenocorticotropic hormone (ACTH)-secreting cells, and to explore the potential clinical application of an inhibitor of Hsp90, 17-N-allylamino-17-demethoxygeldanamycin(17-AAG) in corticotropinomas [also known as “Cushing’s disease” (CD)].

**Methods:**

Culture of mouse pituitary tumor [AtT-20/D16v-F2 (ATCC^®^ CRL-1795™)] cells and human pituitary ACTH-secreting tumor cells were employed. Hepatocellular carcinoma cell line (HLE) was used to evaluate EGFR inhibition by 17-AAG. Cell viability was evaluated using a commercial kit. The ACTH level was measured by a radioimmunoassay. Reverse transcription quantitative polymerase chain reaction (RT-qPCR) was used to measure expression of proopiomelanocortin (POMC) mRNA. Western blotting was done to measure protein levels.

**Results:**

17-AAG suppressed the viability and proliferation, and promoted the apoptosis, of AtT-20/D16v-F2 cells. 17-AAG suppressed the synthesis and secretion of ACTH in AtT-20/D16v-F2 cells and down-regulated POMC transcription. 17-AAG acted in a similar pattern upon treatment with human pituitary ACTH-secreting tumor cells. Inhibition by 17-AAG was stronger in human pituitary ACTH-secreting tumor cells carrying the ubiquitin-specific protease-8 (*USP8*) mutant in comparison with cells carrying wild-type *USP8*.

**Conclusions:**

The HSP90 inhibitor 17-AAG reduced the viability and secretory function of human pituitary ACTH-secreting tumor cells, and tumor cells carrying the *USP8* mutant were more sensitive to 17-AAG than tumor cells carrying wild-type *USP8*. 17-AAG could be a potential treatment option for CD.

## Introduction

Pituitary adenoma is one of the most common tumors appearing at sella. Corticotropinoma accounts for about 2%−6% of pituitary adenomas (1). Corticotropinoma (Cushing disease) is responsible for ~85% of cases of Cushing’s syndrome (2). The clinical manifestations of hypercortisolemia are weight gain, fat redistribution, central obesity, purple striae, ecchymoses, muscle atrophy, and related complications [e.g., hypertension, diabetes mellitus, hyperlipidemia, mental/psychological disorders, osteoporosis, deep-vein thrombosis (2)].

First-line treatment for CD is transsphenoidal surgery, which carries a remission rate of 80%–90% and recurrence of approximately 10–20% (3). Patients with persistent disease can undergo secondary procedure, which carries a remission rate of ≤50% (4). Radiotherapy or medication are options for patients who cannot achieve remission by surgery (5).

Pharmacologic therapy includes drugs that target pituitary tumors, such as dopamine-receptor agonists [e.g., cabergoline (6), octreotide (7)], the adrenal gland [e.g., ketoconazole, metoprolol, metyrapone (5, 8, 9)], or cortisol receptors in peripheral tissues [e.g., mifepristone (10)]. Patients who do not respond to such treatment can undergo bilateral adrenalectomy, which results in permanent adrenal insufficiency (3).

In 2015, we revealed a highly frequent somatic mutation of *USP8* (which encodes ubiquitin-specific protease-8); nearly 60% of CD patients carried this gain-of-function mutation (11). This mutant interfered with the interaction between USP8 and the 14-3-3 motif, resulting in the reduction of the degradation of epidermal growth factor receptors (EGFRs). Therefore, ACTH-secreting adenomas with a *USP8* mutation displayed higher expression of the EGFR, and increased mRNA transcription of proopiomelanocortin (POMC), which is the precursor of ACTH (11, 12). ACTH-secreting adenomas carrying the *USP8* mutation were significantly smaller than genome-wild adenomas, which indicated greater secretion of ACTH (11). Research on primary tumor cells demonstrated that knockdown of USP8 expression or EGFR blockade could inhibit ACTH secretion effectively. Inhibition of expression of USP8 or EGFR could be a potential therapeutic target for patients with corticotropinomas carrying the *USP8* mutation (11, 13).

Heat-shock protein Hsp90 is an important “molecular chaperone” that serves as a key regulator of protein function in eukaryotic cells during stress. Hundreds of “client” proteins interfere with HSP90 and regulate conformational changes in Hsp90 during DNA repair, the immune response, and neurodegenerative changes. Because HSP90 plays an important part in several intracellular processes, it has become a potential target for treatment of various tumor types, as well as neurodegenerative diseases and autoimmune diseases (14).

17-N-allylamino-17-demethoxygeldanamycin (17-AAG) is a derivative of the antibiotic geldanamycin. 17-AAG is an HSP inhibitor, and binds to the N-terminus of HSP90; 17-AAG is being studied as an anti-tumor antibiotic (15). Several studies have shown that HSP90 inhibitors display certain therapeutic effects on immunogenic tumors (e.g., lymphomas, melanomas) and some solid tumors [e.g., liver cancer, breast cancer, non-small-cell lung cancer (16, 17)]. Studies have also suggested that inhibition of HSP90 expression can down-regulate expression of EGFR mutants (18–20) and become potential second-line treatment for non-small-cell lung cancers (21–23).

Based on the previous research of high-frequency mutations USP8 in corticotropinoma, this study tended to explore the regulation of the EGFR pathway by HSP90 involved in the pathogenesis of the tumor, discover potential treatment for Cushing’s disease, and provide evidence for precise treatment.

## Methods

### Cell Culture

Mouse pituitary tumor [AtT-20/D16v-F2 (ATCC^®^ CRL-1795™)] cells (American Type Culture Collection Manassas, VA, USA) were cultured in 75 cm^2^ flasks with Dulbecco’s modified Eagle’s medium (DMEM; Gibco, Billings, MT, USA), 10% fetal bovine serum (Gibco), and 100 U/ml of penicillin–streptomycin (Invitrogen, Carlsbad, CA, USA) in an atmosphere of 5% CO_2_/95% air at 37°C. AtT-20/D16v-F2 cells were incubated with 17-AAG(Cat. No. HY-10211 MedChenExpress Co.) for 72 h before cell assays.

HLE cell line, adherent growing, were cultured in 75 cm^2^ flasks with Dulbecco’s modified Eagle’s medium (DMEM; Gibco, Billings, MT, USA), 10% fetal bovine serum (Gibco), and 100 U/ml of penicillin–streptomycin (Invitrogen, Carlsbad, CA, USA) in an atmosphere of 5% CO_2_/95% air at 37°C. 

Freshly resected human pituitary ACTH-secreting tumors from the Department of Neurosurgery at Huashan Hospital (Shanghai Pituitary Tumor Center, Shanghai, China) were acquired and transferred to 0.5% fetal bovine serum-containing DMEM. Tumor tissue was minced into pieces (1–2 mm) and digested with DMEM containing 0.3% collagenase (Sigma–Aldrich, Saint Louis, MD, USA) and 0.15% hyaluronidase for 60 min at 37°C. The mixture was filtered with a cell strainer to remove undigested tissues and centrifuged at 180 g for 5 min at room temperature. The cell pellet was resuspended in fresh growth medium. Primary tumor cells were incubated with 17-AAG for 48 h before analyses. Clinical manifestations of the patients were showed in **Table 1**.

**Table 1:** The clinical information on the six human pituitary tumors collected.

Patients	Age	Gender	USP8 Mutant	mUFC µg/24H	Serum ACTH pg/ml	Cortisol Circadian Rhythm	Tumor Size cm^3^	Pathology
1#	35	M	+	1293.30	95.1	–	0.4*0.3*0.3	Pituitary ACTHoma
2#	29	F	+	298.52	75.1	–	0.4*0.3*0.3	Pituitary ACTHoma
3#	48	F	+	748.70	50.4	–	1.0*0.5*0.5	Pituitary ACTHoma
4#	45	F	–	341.25	45.7	–	1.0*1.0*1.0	Pituitary ACTHoma
5#	22	F	–	1132.5	25.94	–	1.0*0.75*0.75	Pituitary ACTHoma
6#	31	F	–	486.42	63.05	–	1.0*0.75*0.75	Pituitary ACTHoma

### Colony-Formation Assays

Colony-formation assays were based on clone proliferation. Briefly, AtT-20/D16v-F2 cells were seeded in 6-well plates at low density (~1000 cells per well) The plates were treated 1 μM 17-AAG and DMSO as control, and cultured for 7 days. Then, the plates were washed with phosphate-buffered saline and stained with Crystal Violet. The images of each well were scanned, and individual clones identified. The number of clones that re-generated (each colony >50 cells) was counted to determine colony formation.

### Cell-Viability Assay

AtT-20/D16v-F2 cells were seeded in 96-well plates (Nunc, Roskilde, Denmark) at 10^4^ cell/ml, and treated with 17-AAG with concentration at 1, 2, 5, and 10 μM for 24, 48, 72, and 96 h. Cells incubated with DMSO(control) or a series of 2-fold-diluted concentrations of 17-AAG for 72 h to evaluate IC_50_ Cell viability was measured using the CellTiter-Glo^®^ Luminescent Cell Viability Assay kit according to manufacturer (Promega, Madison, WI, USA) instructions.

### Western Blotting

Cells were lysed in RIPA lysis buffer (Sigma–Aldrich) complemented with a protease inhibitor cocktail (Roche, Basel, Switzerland) to obtain whole-cell lysates. Proteins were separated by electrophoresis on 4%–12% Bis-Tris gels and electroblotted onto polyvinylidene difluoride membranes using the Trans-Blot^®^ Turbo™ Transfer System (Bio-Rad Laboratories, Hercules, CA, USA). Several primary antibodies were used: anti-caspase 3 (Cell Signaling Technology, Danvers, MA, USA), anti-cleaved caspase 3 (Cell Signaling Technology), anti-EGFR (Abcam, Cambridge, UK), anti-phosphorylated extracellular signal-regulated kinase (pERK)1/2 (Cell Signaling Technology) and anti-ERK1/2 (Cell Signaling Technology).

### Reverse Transcription Quantitative Polymerase Chain Reaction

To determine mRNA expression of POMC, RT-qPCR was carried out using SYBR Premix Ex Taq (Tli RNaseH Plus) (Takara Biotechnology, Shiga, Japan). Hypoxanthine-guanine phosphoribosyltransferase (HPRT) was used as an internal control to normalize expression. The following primers (forward and reverse, respectively) were used: 5′-ACCACGGAAAGCAACCTG-3′ and 5′-GTGGCCCATGACGTACTTC-3′ for POMC; 5′-ACCAGTCAACAGGGGACATAAA-3′ and 5′-CTGACCAAGGAAAGCAAAGTCT-3′ for HPRT.

### Sanger Sequencing

Detection of gene mutations was completed by Sanger sequencing。The oligopeptide primers were designed by the Primer3 program. The primers were: Forward: 5'-ATTCACCCACCAACACTGTTCATA-3', Reverse: 5'-TTGTTTTCCCGATTAACTGTTGGA-3'. Dual 384-well GeneAmp PCR System9700 (Applied Biosystems) was used. DNA template 20ng, and 2* Taq PCR Master Mix (Lifefeng Biotech) were added to each system to complete PCR amplification. Sequencing was tested by Big Dye Terminator v.3.1 kit (Applied Biosystems), and analyzed by Chromas Software (Technelysium).

### Radioimmunoassay

We adopted RIA to measure ACTH secreted by primary corticotropinoma tumor cells. Before plasma ACTH measurement, we cultured primary tumor cells in a 6-well plate with a normalized cell count of 10^6 per well. The plasma concentration of ACTH was measured by a sensitive and specific commercial radioimmunoassay, Immulite One (Siemens Medical Solutions, Malvern, PA) The sensitivity of the radioimmunoassay was 4.13 nmol/L for ACTH, and the inter- and intra-assay coefficient of variation was 5%–7% and 6%–7.9%, respectively. The final results are expressed as pictograms per milliliter.

### ELISA

We measured *in-vitro* ACTH from AtT20/D16v-F2 cell line by ELISA. We cultured AtT20/D16v-F2 cells in a 96-well palte with a normalized cell count of 5000 per well, 3 templates for each sample. We used a commercial kit (ALPCO, ACTH ELISA) in this experiment. First configure different concentrations of standards for testing to draw a standard curve. After adding sample to the provided enzyme-labeled well plate has been coated with streptavidin, add biotin-labeled antibody to each well, and then add the enzyme-labeled antibody. Allow 4 h + -30 min reaction time at room temperature, wash the plate 5 times, then add tetramethylbenzidine (color developer), and check the result with a spectrophotometer.

### Statistical Analyses

Statistical analyses were undertaken using SPSS v25.0 (IBM, Armonk, NY, USA). Prism 7.0 (GraphPad, San Diego, CA, USA) was used to prepare graphs. The Student’s *t*-test was used for continuous variables. The Pearson chi-square test was employed for categorical variables. P < 0.05 was considered significant.

## Results

### 17-AAG Inhibits the Viability of AtT-20/D16v-F2 Cells

**Figure 1:**
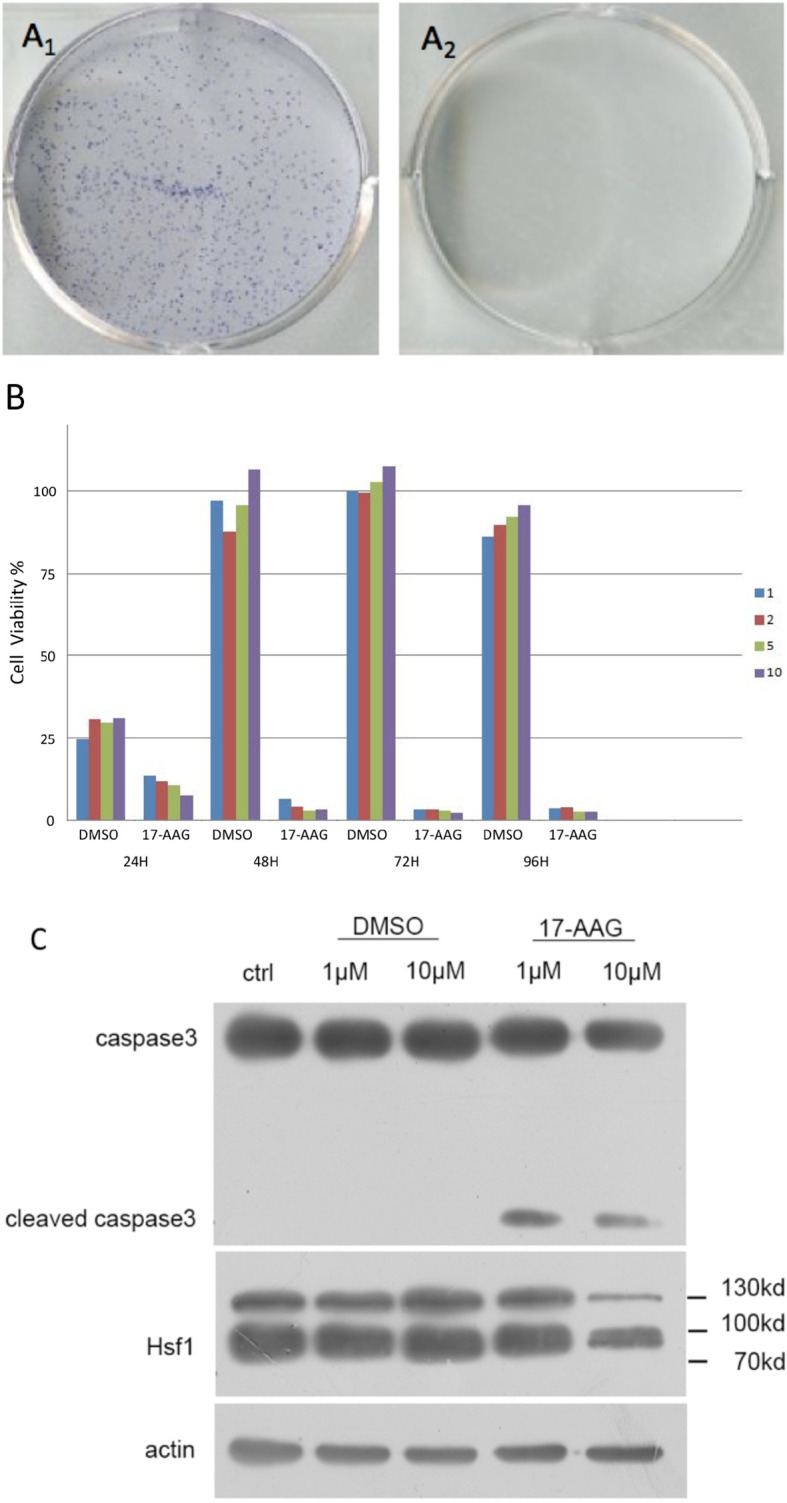
17-AAG inhibits the viability of AtT-20/D16v-F2 cells. In the latter, colony formation was suppressed by 17-AAG (1μM) **(A)**. Caspase-3 expression demonstrated that 17-AAG promoted apoptosis of AtT-20/D16v-F2 cells **(C)**. Proliferation of AtT-20/D16v-F2 cells was suppressed by 17-AAG remarkably in a dose- and time-dependent manner **(B)**. All the experiments were repeated three times with consistent results.

First, we demonstrated that 17-AAG could inhibit cell survival for a long time using colony-formation assays. No colony was formed in the petri dish treated with 17-AAG, whereas the mock petri dish presented with 77.8% colony formation (**Figure 1**).

Caspase-3 expression measured by immunoblotting showed 17-AAG promoted apoptosis of AtT-20/D16v-F2 cells (**Figure 1C**). Caspase-3 expression was decreased in 17-AAG-treated AtT-20/D16v-F2 cells, whereas expression of cleaved caspase-3 was increased, suggesting that 17-AAG activated programmed cell death. Hence, 17-AAG not only inhibited proliferation of AtT-20/D16v-F2 cells, it also promoted their apoptosis.

We treated AtT-20/D16v-F2 cells with 17-AAG (1, 2, 5, and 10 μM) for 24, 48, 72, and 96 h (**Figure1B**). Viability of AtT-20/D16v-F2 cells was suppressed according to drug dose and exposure time. Upon treatment for >72 h, proliferation stopped, so we chose 72 h as the treatment time to measure the half-maximal inhibitory concentration (IC_50_).

### 17-AAG Suppressed of Proliferation of AtT-20/D16v-F2 Cells and ACTH Secretion

**Figure 2:**
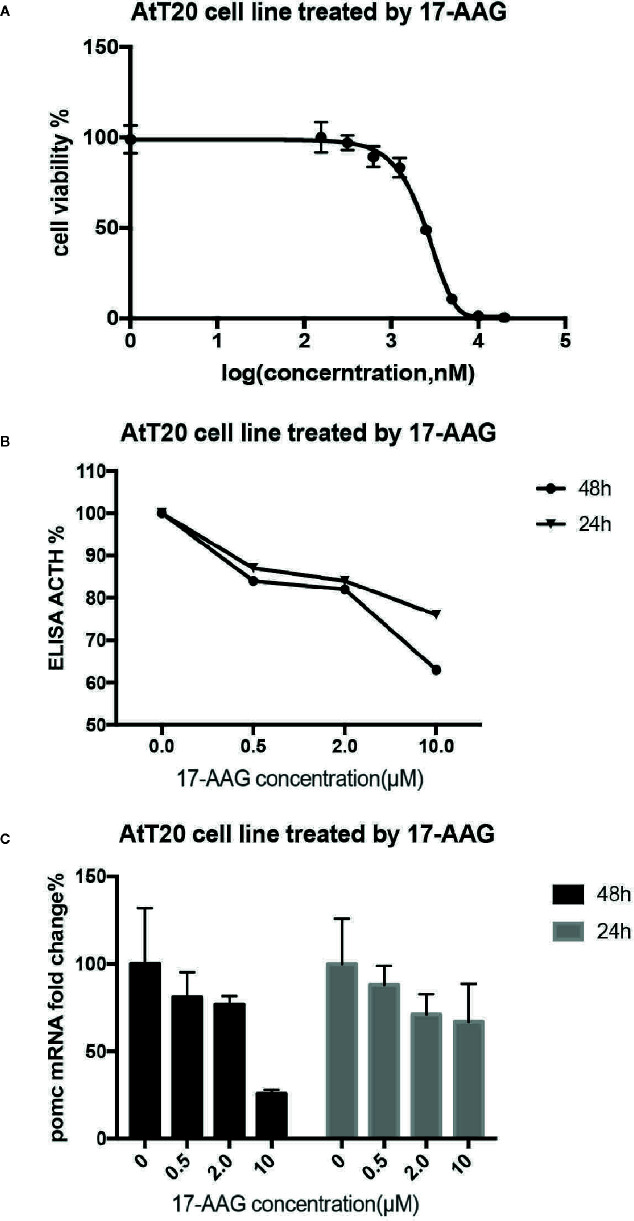
17-AAG suppressed proliferation of AtT-20/D16v-F2 cells and ACTH secretion. The IC_50_ of 17-AAG on AtT-20/D16v-F2 cells was restrained significantly after 17-AAG treatment in a dose-related manner **(B)**. 17-AAG restrained transcription of POMC in AtT-20/D16v-F2 cells and exhibited dose-related effects **(C)**. Three independent experiments were repeated for each result and three replicates were studied in each single experiment.

proliferation of AtT-20/D16v-F2 cells was found to be effectively inhibited by 17-AAG in a dose-dependent manner (**Figure 2A**). AtT-20/D16v-F2 cells were treated for 72 h with a maximum dose of 20 μM of 17-AAG, and their viability was assessed. The IC_50_ was calculated to be 2.33 μM.

AtT-20/D16v-F2 cells were treated with different concentrations of 17-AGG. Cell supernatants were collected for ACTH level measurement(**Figure 2B**). ACTH secretion from AtT-20/D16v-F2 cells was restrained after 17-AAG treatment, and the inhibitory effect of 17-AAG was dose-dependent. ACTH secretion from AtT-20/D16v-F2 cells was impaired more remarkably after 48 h than 24 h, and secretion was similar upon treatment with higher doses of 17-AGG. When treated with a high dosage of 10 μM for 48 h, ACTH level would reduce approximately 40% from baseline.

Further experiments were conducted to investigate the effect of 17-AAG on expression of POMC mRNA in AtT-20/D16v-F2 cells (**Figure 2C**). The latter were treated with different doses of 17-AAG for 24 and 48 h, respectively, and mRNA expression of POMC was determined by RT-qPCR. 17-AAG restrained transcription of POMC in AtT-20/D16v-F2 cells in a dose-related manner. POMC transcription level reduced approximately 75% from baseline when treated with a high dosage of 10 μM for 48 h.

### 17-AAG Inhibited Cell Viability and Secretion of Human Pituitary ACTH-Secreting Tumor Cells

**Figure 3:**
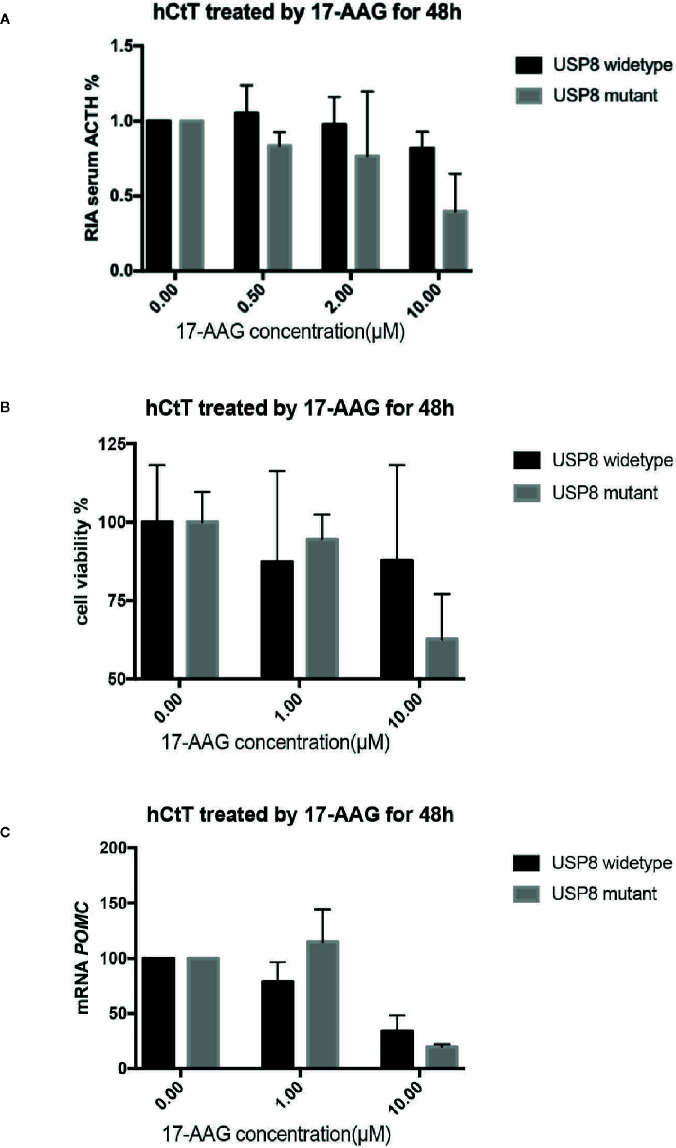
17-AAG inhibited proliferationof primary ACTH-secreting tumor cells and ACTH secretion. Such inhibition was more remarkable in tumor cells carrying the *USP8* mutant. Reduced secretion of ACTH from primary ACTH-secreting tumor cells occurred in a dose-related manner compared with that in the blank control group **(A)**. The viability **(B)** and POMC transcription **(C)** were reduced when primary ACTH-secreting tumor cells were treated with 17-AAG, especially in the *USP8*-mutant group. Three independent experiments were repeated for each result and three replicates were studied in each single experiment.

After 48 h exposure to 17-AAG at dosage of 0.5, 2, and 10 μM, we measured the ACTH concentration in cell supernatants (**Figure 3A**). We found reduced levels of ACTH secretion from primary human ACTH-secreting pituitary tumor cells in a dose-related manner compared with that in the blank control group. In *USP8* mutant group, the ACTH level was suppressed to about 39% from baseline, while in wild type group just to 82% when primary cells treated with 10 μM. Therefore, we examined the mechanism by which 17-AAG may affect ACTH secretion. Cell-viability tests showed that decreased secretion of ACTH might be related to the reduced number of living cells (**Figure 3B**), which suggested that the antitumor effect of 17-AAG partially through inhibition of cytostatic activity. In *USP8* mutant group, the cell viability was suppressed to about 54% from baseline, while in wild type group just to 84% when primary cells treated with 10 μM. Furthermore, qRT-PCR detected reduced transcription of POMC by 17-AAG treatment. (**Figure 3C**). In the *USP8* mutant group, 17-AAG inhibited POMC translation significantly (74% inhibition, p=0.02); While a trend towards reduced POMC expression was noted in the wild type group (63%inhibition, p=0.06), which meant the therapeutic validity manifested on function damage in tumor cells carrying the *USP8* mutant were more sensitive.

### EGFR and Its Downstream Pathway Is Suppressed by 17-AAG in a Model Cell Line

**Figure 4:**
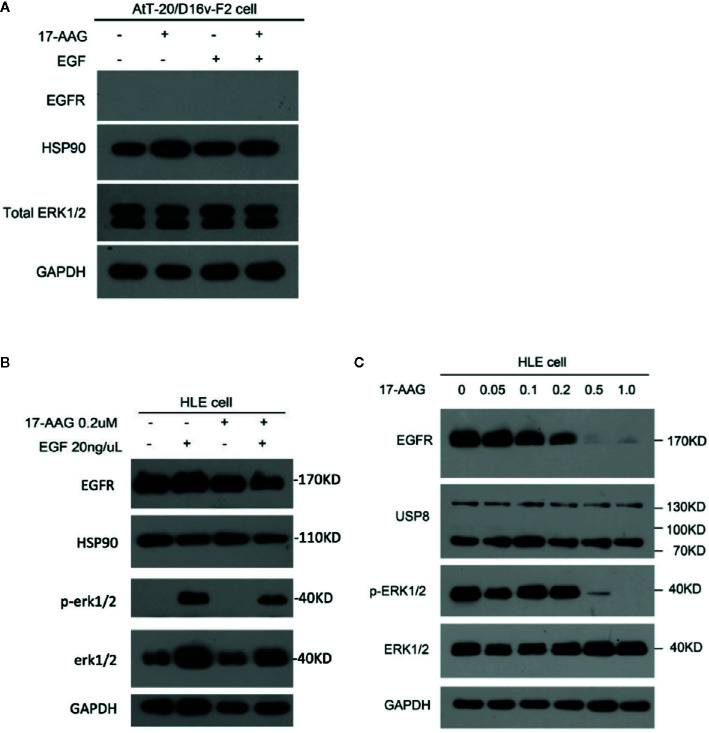
EGFR and its downstream pathway was suppressed by 17-AAG in HLE cells.AtT-20/D16v-F2 cells did not have EGFRS **(A)**, so we used HLE cells instead. Western blotting demonstrated expression of EFGRs and p-ERK1/2 to be down-regulated by 17-AAG treatment **(B, C)**, which suggested that EGFR and its downstream MAPK pathway were suppressed by the HSP90 inhibitor 17-AAG. All the experiments were repeated three times with consistent results.

AtT-20/D16v-F2 cells showed negative EGFR expression (**Figure 4A**), so we used the HLE instead. 17-AAG (0.2 μM) could inhibit EGFR expression in our model cell line. Therefore, we continue to increase the drug concentration. We found that the EGFR expression in model cells decreased after 17-AAG treatment, as well as HSP90. We further used EGF to activated cells, and discovered that the p-ERK1/2 was lower in the 17-AAG treated group compared to the blank control. This indicated that HSP90 inhibition arrested the downstream mitogen-activated protein kinase (MAPK) pathway, which was activated to generate ACTH (**Figure 4B**). From **Figure 4C**, we found that at the dosage of 0.5 nM and 1.0 nM, 17-AAG could limit the inhibition of EGFR expression significantly. After autophosphorylation, EGFR bound directly or indirectly to Grb-2 to activate Ras, then activated the downstream silk/threonine protein kinase Raf, thereby phosphorylating ERK1/2. Deficiency of EGFR expression led to shortage of downstream p-ERK1/2. Therefore, we treated HLE cells with 17-AAG and EGF for further experiment (**Figures 4B, C**). We found that the EGFR expression in model cells decreased after 17-AAG treatment, as well as HSP90. We further used EGF to activated cells, and discovered that the p-ERK1/2 was lower in the 17-AAG treated group compared to the blank control. This indicated that HSP90 inhibition arrested the downstream mitogen-activated protein kinase (MAPK) pathway, which was activated to generate ACTH. At the same time we found, even treated with high concentration of 17-AAG, the restrain to USP8 expression were still slightly. Thus, HSP90 inhibitors were not capable to intervene USP8 expression.

## Discussion

Unlike the dopamine-receptor agonists acting sensitively on prolactinomas, or somatostatin analogs acting on growth hormone-secreting adenomas, pharmacologic options for ACTH-secreting adenomas are limited to drugs targeting pituitary tumors, adrenal glands, or peripheral glucocorticoid receptors. Ketoconazole can inhibit cortisol synthesis, leading to a normal level of cortisol in 50% of patients, but its use is accompanied with hepatotoxicity.

Our research team had revealed a high prevalence (~60%) of *USP8* mutations in ACTH-secreting adenomas (11). This mutation exists only in corticotropinomas, suggesting that USP8 may play an important part in CD pathogenesis. Immunohistochemical studies have confirmed strong overexpression of HSP90 in AtT-20/D16v-F2 cells, as well as the ACTH-secreting tumor tissue (24). 17-AAG inhibit HSP90 function to induce anti-tumor activity.

Studies have revealed the mechanism of USP8-related secretion in ACTH-secreting adenomas (11, 12). Different from genome-wild USP8 Deubiquitinating enzymes(DUBS), *USP8* mutants cannot be phosphorylated or bind to 14-3-3 protein to activate USP8. The function of USP8 is to deubiquitinate molecules and prevent client proteins from degradation. Accumulation of the EGFR causes up-regulation of signaling pathways and thereby accelerates POMC transcription by inducing the deregulation of p27 (25, 26), or degradation of other regulators such as histone deacetylase. Hence, inhibition of USP8 and/or EGFRs represents a potential remedy for CD. Studies in primary tumor cells with USP8 mutants have shown that gefitinib treatment (EGFR inhibitor) can reduce ACTH secretion (11, 13).

When 17-AAG acted upon AtT-20/D16v-F2 cells, it suppressed their colony formation and viability remarkably. Cell proliferation was constrained considerably, with an IC_50_ of 2.33 µM. Connections between apoptosis and ACTH and 17-AAG were found by measurement of caspase-3 expression. Further analyses showed the positive correlation between the 17-AAG concentration and ACTH secretion in AtT-20/D16v-F2 cells was based on the drug dose and exposure time. RT-qPCR also confirmed the conclusion with POMC transcription result. Experiments on AtT-20/D16v-F2 cells suggested that 17-AAG could suppress their viability and ACTH secretion.

Because of the deficiency of EGFR expression in AtT-20/D16v-F2 cells and difficulty in obtaining human corticotropinomas cell line, we used a model cell line HLE which delivered EGFR and human primary tumor cells. In primary ACTH-secreting tumor cells we verified those findings and, interestingly, mutant tumor cells exhibited more sensitivity towards 17-AAG in terms of cell viability and POMC transcription. Expression of EGFRs and p-ERK1/2, which activated in the downstream MAPK pathway, was reduced distinctly by 17-AAG within dosage-related manner. Geldanamycin(GA), a specific inhibitors of the cytosolic chaperone HSP 90, were shown to accelerate degradation of the EGFR (27). GA blocks processing of newly synthesized EGFR. The effects of GA on receptor degradation are mediated by the cytosolic portion of EGFR and could be conferred to the erythropoietin receptor (EPO-R), by employing the respective chimera. But GA didn’t affect stability of newly synthesized EGFR lacking the cytosolic domain (Ex EGFR) (27).However, Geldanamycin has not been used in clinical scenario because of liver toxicity. 17-AAG is a new derivative of geldanamycin that shares its important biological activities but shows less toxicity (28). Hence, EGFR function could be restrained and POMC transcription mediated by the MAPK pathway reduced after inhibition of HSP90 expression. These actions would contribute to tumor-growth inhibition in signaling pathways involving ACTH secretion.

While, in the context of *USP8* mutated, the deregulation of EGFR led to improve MAPK signaling and subsequently promoted POMC transcription, which further result to the oversecretion of ACTH. *In-vitro* model cell experiments also confirmed that HSP90 inhibitor could significantly reduce the expression level of EGFR and lower down-regulate the MAPK pathway. We believed that overexpressed EGFR provided greater targets for HSP90 inhibitors, therefore 17-AAG manifested a more significant effect on *USP8* mutant tumor cells.

HSP90, as a molecular chaperone, plays a key role in the conformational maturation of oncogenic signaling proteins. HSP90 inhibitors selectively kill cancer cells compared to normal cells, and have also demonstrated antitumor effects towards lymphomas, melanomas, as well as some solid tumors (e.g., breast cancer, non-small-cell lung cancer) (16, 17). To date the majority of pharmaceutical research and development focused on targeting the N-domain ATP binding site of HSP90, C-terminal of the protein on the heels. Silibinin, as well as novobiocin, were the prototypic inhibitors that interact at the C-domain site. And the therapeutic effect in cancer had been demonstrated. One conceivable benefit of these medication is that certain C-terminal inhibitors appear to have weaker HSF1 activation capacity. The ongoing study support further optimization of medicinal chemistry and preclinical evaluation of C-terminal Hsp90 inhibitors. The four Hsp90 subtypes (Hsp90α and Hsp90β in the cytoplasm and nucleus, the relative importance of GRP94 and TRAP1 in the endoplasmic reticulum in cancer) and mitochondria were still under research (29). Our present study provides a foundation for potential clinical treatment of CD by HSP90 inhibitor. Further research will continue to build animal models to improve related *in-vivo* experiments. It will provide more detailed research basis for the clinical application of HSP90 inhibitors in the treatment of Cushing’s disease.

## Conclusions

The HSP90 inhibitor 17-AAG reduced the viability and secretory function of human pituitary ACTH-secreting tumor cells, and tumor cells carrying the *USP8* mutant were more sensitive to 17-AAG than tumor cells carrying wild-type *USP8*. 17-AAG could be potential clinical treatment for CD.

## Data Availability Statement

The original contributions presented in the study are included in the article/supplementary material. Further inquiries can be directed to the corresponding authors.

## Ethics Statement

Written informed consent was obtained from the individual(s) for the publication of any potentially identifiable images or data included in this article.

## Author Contributions

Conception or design of the work: ZM and XZ. Financial support: ZM, QZ, and YZ. Surgical specimen recruitment: NQ, YZ, XS, XZ, ZM. Laboratory Practice: YS, CJ. Data analysis and interpretation: YS, ZM, QZ. Drafting the article: YS, CJ. Critical revision of the article: ZM, XZ. All authors contributed to the article and approved the submitted version.

## Funding

The research was supported by the National Science Fund for Distinguished Young Scholars (81725011), Clinical Research Plan of SHDC(SHDC2020CR2004A) to YZ; the National Natural Science Funds of China (81702467), Shanghai Sailing program (17YF1401500) to ZM; National Natural Science Funds of China (81802495), Shanghai Sailing program (18YF1403400) to QZ.

## Conflict of Interest

The authors declare that the research was conducted in the absence of any commercial or financial relationships that could be construed as a potential conflict of interest.
